# Computed Tomography Structural Lung Changes in Discordant Airflow Limitation

**DOI:** 10.1371/journal.pone.0065177

**Published:** 2013-06-13

**Authors:** Firdaus A. A. Mohamed Hoesein, Pim A. de Jong, Jan-Willem J. Lammers, Willem PThM Mali, Michael Schmidt, Harry J. de Koning, Carlijn van der Aalst, Matthijs Oudkerk, Rozemarijn Vliegenthart, Bram van Ginneken, Eva M. van Rikxoort, Pieter Zanen

**Affiliations:** 1 Department of Respiratory Medicine, Division of Heart & Lungs, University Medical Center Utrecht, Utrecht, The Netherlands; 2 Department of Radiology, University Medical Center Utrecht, Utrecht, The Netherlands; 3 Fraunhofer MEVIS, Institute for Medical Image Computing, Bremen, Germany; 4 Department of Public Health, Erasmus MC, Rotterdam, The Netherlands; 5 Center for Medical Imaging – North East Netherlands, University of Groningen, University Medical Center Groningen, Groningen, The Netherlands; 6 Department of Radiology, University of Groningen, University Medical Center Groningen, Groningen, The Netherlands; 7 Department of Radiology, Radboud University Nijmegen Medical Centre, Nijmegen, The Netherlands; University of Washington School of Medicine, United States of America

## Abstract

**Background:**

There is increasing evidence that structural lung changes may be present before the occurrence of airflow limitation as assessed by spirometry. This study investigated the prevalence of computed tomography (CT) quantified emphysema, airway wall thickening and gas trapping according to classification of airflow limitation (FEV_1_/FVC <70% and/or < the lower limit of normal (LLN)) in (heavy) smokers.

**Methods:**

A total number of 1,140 male former and current smokers participating in a lung cancer screenings trial (NELSON) were included and underwent chest CT scanning and spirometry. Emphysema was quantified by the 15^th^ percentile, air way wall thickening by the square root of wall area for a theoretical airway with 10mm lumen perimeter (Pi10) and gas trapping by the mean lung density expiratory/inspiratory (E/I)-ratio. Participants were classified by entry FEV_1_/FVC: group 1>70%; group 2<70% but >LLN; and group 3<LLN. 32 restricted subjects, i.e. FEV_1_/FVC >70% but FEV_1_ <80% predicted, were excluded. Multivariate regression analysis correcting for covariates was used to asses the extent of emphysema, airway wall thickening and gas trapping according to three groups of airflow limitation.

**Results:**

Mean (standard deviation) age was 62.5 (5.2) years and packyears smoked was 41.0 (18.0). Group 2 subjects when compared to group 1 had a significantly lower 15^th^ percentile, −920.6 HU versus −912.2 HU; a higher Pi10, 2.87 mm versus 2.57 mm; and a higher E/I-ratio, 88.6% versus 85.6% (all p<0.001).

**Conclusion:**

Subjects with an FEV_1_/FVC<70%, but above the LLN, have a significant greater degree of structural lung changes on CT compared to subjects without airflow limitation.

## Introduction

Chronic obstructive pulmonary disease (COPD) is one of the important causes of morbidity and mortality worldwide and its mortality rates are still rising. [1] Paradoxically, it still is being under diagnosed. [2] COPD is characterized by the presence of airflow limitation, i.e. when the forced vital capacity (FVC) to the forced expiratory volume in one second (FEV_1_) -ratio is below a predefined threshold and currently spirometry is used to diagnose COPD. [3]

The airflow limitation is the result of several structural changes in the lung like destruction of airway parenchyma (emphysema) and small airway disease (gas trapping and airway wall thickening). [4] Computed tomography (CT) of the chest is used to quantify the extent of these structural changes. [5] Assessing the degree of these structural changes can give a better insight in the background of airflow limitation in individual subjects and assess the heterogeneity of the disease. There is evidence that these structural changes already are present before airflow limitation is present.

While the Global Initiative for Chronic Obstructive Lung Disease (GOLD) propose a fixed value of FEV_1_/FVC <70% as cut-off for diagnosing airflow limitation others advocate the lower limit of normal (LLN). [3] Both methods have their merits and flaws and currently no there is no consensus on which to use. [6] Unfortunately, in the absence of a real gold standard of COPD a consensus is an ideal state not to be reached soon. [7].

Opponents of the fixed ratio believe that a ratio of 70% results in overdiagnosis. In this study we therefore examined the degree of structural changes in the lung, emphysema, airway wall thickness and gas trapping, by CT in a cohort of relatively healthy male smokers according to their FEV_1_/FVC. Subjects were classified as having no airflow limitation (FEV_1_/FVC >70%), in-between (FEV_1_/FVC <70%, but >LLN) and airflow limitation (FEV_1_/FVC <LLN). We hypothesized that the in-between group had significantly more structural airway changes on CT than those without airflow limitation.

## Methods

### Ethics Statement

The ethics committees of the involving hospitals (University Medical Center Utrecht and University Medical Center Groningen, the Netherlands, IRB approval number 03/040) as well as the Dutch Ministry of Health approved the study. The NELSON trial is registered at www.trialregister.nlwith trial number ISRCTN63545820.

### Subjects

This study was conducted as a sub study of the Dutch and Belgium Lung Cancer Screening Trial (NELSON trial – ISRCTN63545820, registered at www.trialregister.nl). Details of the selection procedure have previously been described. [8] In short, participants in the lung cancer screening trial were 50–75 year old current or former smokers with a smoking history of at least 16 cigarettes/day for 25 years or at least 11 cigarettes/day for 30 years (>16.5 pack-years). [9] Former smokers should not have quitted for more than 10 years at inclusion. Detailed smoking characteristics and symptoms were obtained through a questionnaire. The questionnaire contained the following question on respiratory symptoms: do you have experienced the following symptoms cough, sputum expectoration, wheezing or dyspnea for at least 3 months during the past year, even when you did not have a cold? To further study COPD an expiratory acquisition was added to the screening protocol in the University Medical Center Utrecht, the Netherlands. The NELSON trial was approved by the Ministry of Health of The Netherlands and the institutional ethical review board. Written informed consent was obtained in all screening trial participants.

### Pulmonary function testing

Details on the pulmonary function tests (PFT) have been reported in detail before and included spirometry and body plethysmography. [10] PFT were obtained according to European Respiratory Society/American Thoracic Society (ERS/ATS) guidelines and was performed with ZAN equipment (ZAN Messgeräte GmbH, Germany). [11] No broncho dilatation was applied. Measurements include forced expiratory volume in one second (FEV_1_), forced vital capacity (FVC), mid expiratory flow at 50% of FVC (MEF_50_), residual volume (RV), total lung capacity (TLC) and the transfer coefficient for carbon monoxide (Kco) as a measure of lung diffusion capacity. Lower limits of normal were calculated using the reference equations of the European Community of Coal and Steel (ECCS). [12] RV/TLC was expressed as a percentage.

### CT scanning

The CT protocol has been described in detail before. [8], [13] In short, all CTs were performed without intravenous contrast injection, and obtained with 16×0.75 mm collimation on the same scanner (Brilliance 16P, Philips Medical Systems, USA). Volumetric inspiratory and end-expiratory CT scans were obtained after standardized breathing instructions in all subjects. Subjects weighing 80 kg or less were scanned with 120 kVp at 30 mAs for inspiratory acquisition and 90 kVp at 20 mAs for expiratory acquisition (total effective dose 0.98 and 0.27 mSv, respectively). Axial images were reconstructed from lung bases to lung apices at a slice thickness of 1.0 mm at 0.7 mm increment, using a smoothed reconstruction filter (B-filter, Philips).

### Quantitative CT assessment of the lung parenchyma

As previously described, briefly, the lungs were automatically segmented from the chest wall, airways and mediastinum using dedicated software. [14] A noise reduction filter was applied to decrease the influence of noise on the quantitative measurements. [15] CT emphysema was defined as the percentage of voxels below Hounsfield Unit (HU) −950 and the 15^th^ percentile (Perc15). The Perc15 is the HU number below which 15% of voxels are distributed and a lower Perc15, i.e. more close to −1000 HU, points at more emphysema. Because the percentage of voxels below HU −950 is not normally distributed it is log-transformed (log950%). CT gastrapping was defined as the expiratory mean lung density in HU divided by the inspiratory mean lung density expressed as a percentage (E/I-ratio).

### Quantitative CT assessment of large airways

The airway lumen was automatically segmented. [16] Airway cross-sections are defined perpendicular to the local airway direction at a spacing of 1mm across all airway centerlines and inner and outer airway wall borders are segmented for each of these cross-sections. [17] Obviously failed airway wall segmentations and cross-sections were automatically discarded from further analysis. A linear regression of the square root of wall area versus the lumen perimeter was calculated for the remaining cross-sections and the square root of wall area for a theoretical airway with 10mm lumen perimeter (Pi10) was calculated which was used as measurement of airway wall thickness. [18] For each CT scan a random selection of cross-sections of the detected airway wall borders was visually inspected to verify measurement accuracy. Cases with unsatisfactory results were left out of the airway analysis.

### Data analysis

Descriptive statistics are presented as mean ± standard deviation (SD) for normally distributed variables and as median and inter-quartile range (IQR) for non-normally distributed variables. Distribution of normality was visually checked by probability plots. Univariate analyses were done with Students' T-tests and chi-squared tests, respectively for normal and non-normal distributed variables. The group without airflow limitation (i.e. FEV_1_/FVC >70%) was used as reference. The degree of structural lung changes at CT (emphysema, gas trapping and airway wall thickening) were analyzed by analysis of covariance with class of airflow limitation (FEV_1_/FVC >70%; <70% and >LLN; and <LLN) as main explanatory variable. Again the group FEV_1_/FC>70% was used as reference. Adjustments were made for age, height, BMI, packyears, smoking status (current or former) and presence of respiratory symptoms. A p-value of <0.05 was considered significant. All statistical analyses were performed using SPSS 18 (SPSS, Chicago, USA).

## Results

### Demographics

Subjects' demographics for the total population and according to airflow limitation, i.e. FEV_1_/FVC >70%; <70%, but >LLN, and <LLN, are presented in Table 1. A number of 1,140 males with were included. Of these 1,140 subjects, 32 had a restrictive lung function pattern, i.e. FEV_1_/FVC > 70% but FEV_1_ <80% predicted, which were excluded resulting in 1,108 subjects included in the current study. Mean (SD) age was 60.4 (19.9) years and mean (SD) packyears was 41.0 (18.0).Approximately half had quit smoking (47.2%). The majority had no airflow limitation according to either the fixed value of FEV_1_/FVC <70% or <LLN (671, 61%). A number of 216 subjects had an FEV_1_/FVC <70%, but >LLN, and 221 subjects had airflow limitation according to both thresholds.

**Table 1:** Baseline demographics, clinical variables and pulmonary function for the total population and stratified by classification of airflow limitation.

	Total, n = 1,108	FEV1/FVC >70%, n = 671	FEV1/FVC <70%, but>LLN, n = 216	FEV1/FVC <70%, and <LLN, n = 221
	Mean (SD)	Mean (SD)	Mean (SD)	Mean (SD)
**Age [years]**	62.5 (5.2)	62.1 (5.1)	63. 7 (5.6)^∧^	62.8 (5.4)^∧^
**Packyears**	41.0 (18.0)	39.9 (17. 7)	41.7 (17.6)^∧^	43.7 (19.3)^#^
**Current smoker, % (n)**	52,8 (585)	49,2 (330)	54,2 (117)^#^	65,6 (145)^#^
**Weight [kilo]**	86.3 (13.1)	88.2 (12. 9)	84.4 (11.9)^#^	82.0 (13.8)^#^
**Height [centimeters]**	178. 5 (6.6)	178.4 (6.4)	178.5 (6.8)	178.5 (7.03)
BMI [kg*m^−2^]	27,1 (3,6)	27. 7 (3.5)	26.5 (3.3)^∧^	25.8 (3.7)^#^
**FEV_1_ % pred**	94. 8 (17.6)	103.9 (12.4)	92.0 (12.7)^#^	75.0 (17.5)^#^
**FEV_1_/FVC %**	70. 9 (9.3)	76.5 (4.4)	67.4 (1. 9)^#^	56.4 (7.9)^#^
**MEF_50_ % pred**	68.8 (29.3)	84.9 (23.9)	53.5 (10.3)^#^	32.6 (12.5)^#^
**RV/TLC %**	35.9 (8.4)	34.2 (7.3)	35.2 (8.1)^∧^	41.0 (9. 5)^#^
**TLC %**	104.7 (14.2)	100.8 (12.8)	107. 8 (13.9)^#^	113.5 (13.5)^#^
**RV %**	111.4 (35.2)	101.6 (8.0)	111.2 (33.2)^#^	136.7 (40.9)^#^
**Kco [mmol/min/kPa/l]**	87.1 (17.4)	92.2 (15.0)	81.3 (15.7)^#^	77. 7 (19.3)^#^
**TLco [mmol/min/kPa]**	8.4 (1.9)	8.8 (1.8)	8.0 (2.0)^∧^	7.4 (2.1)
**Cough, % (n)**	28.4 (315)	23.4 (154)	31.0 (67)^#^	44.8 (99)^#^
**mucus, % (n)**	26.3 (290)	21.9 (147)	25.9 (56)^∧^	40.3 (89)^#^
**Dyspnea, % (n)**	24.7 (272)	20.4 (137)	21.8 (47)^#^	42.5 (94)^#^
**Wheezing, % (n)**	18.3 (202)	14.3 (96)	18.1 (39)^#^	33.5 (74)^#^

Univariate analysis with the group FEV_1_/FVC >70% as reference.?p <0.05- >0.001^ #^ p<0.001

### Lung function and clinical variables

Subjects without airflow limitation had smoked less packyears and more likely had quit smoking, p<0.001. BMI was significantly lower in subjects with airflow limitation according to either threshold of FEV_1_/FVC, all p<0.001. Respiratory symptoms were significantly more prevalent in subjects with an FEV_1_/FVC <70%, but >LLN and <LLN compared to subjects without airflow limitation, all p<0.001. Subjects with a FEV_1_/FVC <70%, but >LLN had a significantly lower lung diffusion capacity compared to those without airflow limitation, p = 0.001. See Table 1.

### Structural CT changes

The degree of emphysema, airway wall thickening and gas trapping for the total population and according to airflow limitation classification are provided in Table 2. In univariate analysis subjects with a FEV_1_/FVC <70%, but >LLN had a lower Perc15, higher Pi10 and higher E/I-ratios measure compared to those without airflow limitation, p<0.001 (Table 2).

**Table 2:** Degree of structural CT changes (emphysema. airway wall thickening and gas trapping) for the total population and stratified by classification of airflow limitation.

	Total, n = 1,108	FEV1/FVC >70%, n = 671	FEV1/FVC <70%, but>LLN, n = 216	FEV1/FVC <70%, and <LLN, n = 221
	Mean (SD)	Mean (SD)	Mean (SD)	Mean (SD)
**15th percentile**	−908.1 (18.9)	−902.6 (17.9)	−912.4 (15. 5)^#^	−921.1 (18.6)^#^
**log950%**	−0.22 (1.14)	−0.61 (0.93)	0.21 (0.95)^#^	0.76 (1.23)^#^
**E/I-ratio%**	83.70 (6.2)	81.67 (5.9)	85.53 (4.9)^#^	88.73 (4.7)^#^
**Pi10 [millimeters]**	2.43 (0.51)	2.28 (0.42)	2.51 (0.49)^#^	2.79 (0.56)^#^

Univariate analysis with the group FEV_1_/FVC >70% as reference.^ #^ p<0.001. Log950% =  log-transformed percentage of voxels below HU -950. E/I-ratio%  =  expiratory mean lung density in HU divided by the inspiratory mean lung density in HU expressed as a percentage. Pi10 =  the square root of wall area for a theoretical airway with 10mm lumen perimeter.

Also in the multivariate analysis with correction for age, height, BMI, packyears, smoking status and presence of respiratory symptoms a difference in emphysema, airway wall thickening and gas trapping remained between subjects without airflow limitation and those with a FEV1/FVC <70%, but >LLN, all p<0.001. Subjects with an FEV_1_/FVC<70%, but >LLN had a mean (95% confidence interval) 8.1 (−10.9–−5.3) lower Perc15, a 0.22 mm (0.14–0.29) higher Pi10 and a 3.25% (2.32–4.27) higher E/I-ratio compared to subjects without airflow limitation, see Table 3.

**Table 3:** Multivariate analysis of mean (95% confidence interval) differences in structural CT changes (emphysema. airway wall thickening and gas trapping) according to classification of airflow limitation correcting for age, height, BMI, packyears and respiratory symptoms.

	FEV_1/_FVC <70%, but>LLN		FEV_1_/FVC <70%, and <LLN
**15th percentile**	−8.1 (−10.9–−5.3)^#^	**15th percentile**	−16.45 (−19.36–−13.5)^ #^
**log950%**	0.58 (0.43–0.73)^#^	**log950%**	1.37 (1.25–1.56)^#^
**E/I-ratio%**	3.25 (2.32–4.27)^#^	**E/I-ratio%**	6.27 (5.32–7.22)^#^
**Pi10 [millimeters]**	0.22 (0.14–0.29)^#^	**Pi10 [millimeters]**	0.51 (0.43–0.59)^#^

The group FEV_1_/FVC >70% was used as reference. ^#^ p<0.001 Log950% =  log-transformed percentage of voxels below HU -950. E/I-ratio% =  expiratory mean lung density in HU divided by the inspiratory mean lung density in HU expressed as a percentage. Pi10 =  the square root of wall area for a theoretical airway with 10 mm lumen perimeter.

In an additional analysis airway wall thickness and gas trapping measures were added to the analysis of covariance for analyzing differences in emphysema between the three groups of airflow limitation. Consequently, emphysema and gastrapping measures were also added to the analysis for differences in airway wall thickness, and emphysema and airway wall thickening were added to the analysis for differences in gas trapping. Again, subjects with a FEV_1_/FVC <70%, but >LLN had more emphysema, airway wall thickening and gas trapping on CT than those without airflow limitation, all p<0.001.

## Discussion

In this study we showed that former and current smokers with an FEV_1_/FVC below 70%, but above the LLN, have significantly lower diffusion capacity, more emphysema, airway wall thickening and gas trapping on CT than those without airflow limitation, even after extensive correction for confounders.

In the current debate on the most appropriate threshold of FEV_1_/FVC for diagnosing airflow limitation the major problem is in those subjects with indeterminate outcomes, i.e. affected according to the fixed threshold but not-affected according to the LLN. Therefore the current study population of relatively healthy, but former and current smokers is well-suited as large number of in-between subjects were included. Those with evident airflow limitation obviously do not form a problem. In a primary care population presenting with chronic cough it has been shown that the fixed value of 70% has better diagnostic values than the LLN in diagnosing COPD. [19] In that study a panel diagnosis of COPD was used as the gold standard taking in account the FEV_1_/FVC value with other relevant clinical factors.

In theory, the most appropriate threshold for FEV_1_/FVC in diagnosing COPD should have the highest sensitivity and specificity as possible. Unfortunately, without a real gold standard for COPD calculating these numbers is not possible and a true comparison of the fixed value and LLN are not possible. We know from studies that using the 70% value diagnoses a larger number of subjects with COPD than when using the LLN. [20], [21] Nonetheless, when choosing a threshold of airflow limitation it at the least should discriminate between subjects with the structural lung changes (emphysema, airway wall thickening) causing the airflow limitation and subjects without.

The results of this study show that sole use of spirometry to diagnose COPD is not ideal. COPD is a complex disease resulting from multiple structural lung changes which not all are caught entirely by the FEV_1_/FVC. Airflow limitation in COPD is caused by two main sites in the lungs; the small airways and the lung parenchyma. [4] In small airways disease the resistance is increased causing airflow limitation. Emphysematous lung parenchyma destructions decrease the lung compliance, i.e. elastic recoil force, which also causes airflow limitation. Both entities usually to a greater or lesser extent coincide. Quantitative chest CT has shown to be a promising tool in detection and quantification of both small airway disease and emphysema. In the current study we have used quantitative CT to show that also in subjects regarded as affect by the 70% criterion, but not affected by the LLN criterion, measures of small airway disease and emphysema are significantly higher compared to healthy subjects.

The in-between subjects in this study had significantly more structural lung changes than those without airflow limitation. This is in concurrence with results from Mannino et al. showing that participants of the Third National Health and Nutrition Examination Survey (NHANES III) with an FEV_1_/FVC <70% but >LLN, comparable to the in-between group in our study, had significantly higher rates of hospitalization and mortality. [22] Another large study reported that the in-between group had a significantly larger consumption of health-care resources. [23] In addition to pulmonary manifestations, it has been reported that participants of the population based Burden of Obstructive Lung Disease (BOLD) study with an FEV1/FVC <70% but >LLN have higher degrees of relevant co morbidities. [24] Yet another study showed that subjects in the in-between group had a worse self-reported quality of life. [25] Taken together with the results of the current study it at the least is questionable whether ignoring patients with an in-between FEV_1_/FVC ratio is acceptable.

Importantly, participants with airflow limitation according to either the fixed value of 70% or the LLN had not only more structural CT changes but also significantly lower diffusion testing outcomes. Our data confirms previous physiological findings on pulmonary gas exchange abnormalities in subjects with mild COPD. [26], [27] Our data support the findings that in mild lung function abnormalities not only lung mechanics are affected, but also pulmonary gas exchange. [27], [28].

To our knowledge, no studies yet assessed the difference in structural lung changes according to classification of airflow limitation in current and former smokers. The strength of the current study is detailed characterization of CT quantified structural lung changes (emphysema, airway wall thickness and gas trapping). One of the limitations of the current study is the use of pre-bronchodilator spirometry in the absence of post-bronchodilator values. This may have resulted in a higher percentage of participants with airflow limitation that actually could be lower. There is however evidence that most subjects will change from limitation status based on non-significant changes in lung function parameters. [20] Secondly, no females were included which is especially unfortunate because the incidence of COPD is rising in females. A previous study showed that females generally have less emphysema and airway wall thickening when compared to males. [29] Future studies should also aim to include females to elucidate whether there is a difference in the presence of structural lung changes according to airflow limitation classification. Finally, only relatively healthy smokers, with no or mild COPD, were included, and no participants with moderate to severe COPD. However, these relatively healthy smokers are the ones most at interest for the assessment of early structural lung changes, some which not yet show lung function abnormalities.

In conclusion, our study showed that relatively healthy (heavy) former and current smokers with an FEV_1_/FVC <70%, but above the LLN, already have a lower pulmonary diffusion capacity and show significantly more structural lung changes on CT compared to subjects without airflow limitation. The current observations at the least questions the notion that using the 70% threshold of FEV_1_/FVC for diagnosing airflow limitation is inappropriate and is causing an over diagnosis of COPD.

## References

[1] Mannino DM, Buist AS (2007) Global burden of COPD: risk factors, prevalence, and future trends. Lancet 370(9589): 765–73. 17765526 10.1016/S0140-6736(07)61380-4

[2] Jordan RE, Lam KH, Cheng KK, Miller MR, Marsh JL, et al (2010) Case finding for chronic obstructive pulmonary disease: a model for optimising a targeted approach. Thorax 65(6): 492–8. 20522845 10.1136/thx.2009.129395 PMC2892752

[3] Vestbo J, Hurd SS, Agusti AG, Jones PW, Vogelmeier C, et al.. (2012) Global Strategy for the Diagnosis, Management and Prevention of Chronic Obstructive Pulmonary Disease, GOLD Executive Summary. Am J Respir Crit Care Med Epub ahead of print. doi: 10.1164/rccm.201204–0596. 10.1164/rccm.201204-0596PP 22878278

[4] Hogg JC (2004) Pathophysiology of airflow limitation in chronic obstructive pulmonary disease. Lancet 364(9435): 709–21. 15325838 10.1016/S0140-6736(04)16900-6

[5] Hackx M, Bankier AA, Gevenois PA (2012) Chronic Obstructive Pulmonary Disease: CT Quantification of Airways Disease. Radiology 265(1): 34–48. 22993219 10.1148/radiol.12111270

[6] Mohamed Hoesein FAA, Zanen P, Lammers JJ (2011) Lower limit of normal or FEV1/FVC <0.70 in diagnosing COPD: an evidence-based review. Respir Med 105(6): 907–15. 21295958 10.1016/j.rmed.2011.01.008

[7] Celli BR, Halbert RJ (2010) Point: should we abandon FEV1/FVC 0.70 to detect airway Ostruction? No. Chest 138(5): 1037–40. 10.1378/chest.10-2049 21051393

[8] Mets OM, Buckens CFM, Zanen P, Isgum I, van Ginneken B, et al (2011) Identification of chronic obstructive pulmonary disease in lung cancer screening computed tomographic scans. JAMA 306(16): 1775–81. 22028353 10.1001/jama.2011.1531

[9] van Iersel CA, Koning HJ de, Draisma G, Mali WPTM, Scholten ET, et al (2007) Risk-based selection from the general population in a screening trial: selection criteria, recruitment and power for the Dutch-Belgian randomised lung cancer multi-slice CT screening trial (NELSON). Int J Cancer 120(4): 868–74. 17131307 10.1002/ijc.22134

[10] Mohamed Hoesein FAA, Zanen P, van Ginneken B, van Klaveren RJ, Lammers JJ (2011) Association of the transfer coefficient of the lung for carbon monoxide with emphysema progression in male smokers. Eur Respir J 38(5): 1012–8. 21565924 10.1183/09031936.00050711

[11] Miller MR, Crapo R, Hankinson J, Brusasco V, Burgos F, et al (2005) General considerations for lung function testing. Eur Respir J 26(1): 153–61. 15994402 10.1183/09031936.05.00034505

[12] Quanjer PH, Tammeling GJ, Cotes JE, Pedersen OF, Peslin R, et al.. (1993) Lung volumes and forced ventilatory flows. Report Working Party Standardization of Lung Function Tests, European Community for Steel and Coal. Official Statement of the European Respiratory Society. Eur Respir J Suppl 16: 5–40. 8499054

[13] Mohamed Hoesein FAA, Hoop B de, Zanen P, Gietema H, Kruitwagen CLJJ, et al (2011) CT-quantified emphysema in male heavy smokers: association with lung function decline. Thorax 66(9): 782–7. 21474499 10.1136/thx.2010.145995

[14] Rikxoort van EM, Hoop B de, Viergever MA, Prokop M, Ginneken van B (2009) Automatic lung segmentation from thoracic computed tomography scans using a hybrid approach with error detection. Med Phys 36(7): 2934–47. 19673192 10.1118/1.3147146

[15] Schilham AMR, van Ginneken B, Gietema H, Prokop M (2006) Local noise weighted filtering for emphysema scoring of low-dose CT images. IEEE Trans Med Imaging 25(4): 451–63. 16608060 10.1109/TMI.2006.871545

[16] Rikxoort van EM, Baggerman W, Ginneken van B (2009) Automatic segmentation of the airway tree from thoracic CT scans using a. multi-threshold approach. The Second International Workshop on Pulmonary Image Analysis 341–9.

[17] Schmidt M, Kuhnigk J, Krass S, Owsijewitsch M, Hoop B de, et al.. (2010) Medical Imaging: Computer-Aided Diagnosis. Proceedings of SPIE: SPIE Vol. 7624.

[18] Patel BD, Coxson HO, Pillai SG, Agusti AGN, Calverley PMA, et al (2008) Airway wall thickening and emphysema show independent familial aggregation in chronic obstructive pulmonary disease. Am J Respir Crit Care Med 178(5): 500–5. 18565956 10.1164/rccm.200801-059OC

[19] Mohamed Hoesein FAA, Zanen P, Sachs APE, Verheij TJM, Lammers JJ, et al (2012) Spirometric thresholds for diagnosing COPD: 0.70 or LLN, pre- or post-dilator values? COPD 9(4): 338–43. 22489910 10.3109/15412555.2012.667851

[20] Prentice HA, Mannino DM, Caldwell GG, Bush HM (2010) Significant bronchodilator responsiveness and “reversibility” in a population sample. COPD 7(5): 323–30. 20854046 10.3109/15412555.2010.510161

[21] Bednarek M, Maciejewski J, Wozniak M, Kuca P, Zielinski J (2008) Prevalence, severity and underdiagnosis of COPD in the primary care setting. Thorax 63(5): 402–7. 18234906 10.1136/thx.2007.085456

[22] Mannino DM (2012) Diaz-Guzman (2012) Interpreting lung function data using 80% predicted and fixed thresholds identifies patients at increased risk of mortality. Chest 141(1): 73–80. 21659434 10.1378/chest.11-0797

[23] Izquierdo Alonso JL, Lucas Ramos P de, Rodríguez Glez-Moro JM (2012) El uso del límite inferior de la normalidad como criterio de EPOC excluye pacientes con elevada morbilidad y alto consumo de recursos sanitarios. Archivos de Bronconeumología 48(7): 223–8.22480962

[24] Danielsson P, Ólafsdóttir IS, Benediktsdóttir B, Gíslason T, Janson C (2012) The prevalence of chronic obstructive pulmonary disease in Uppsala, Sweden – the Burden of Obstructive Lung Disease (BOLD) study: cross-sectional population-based study. The Clinical Respiratory Journal 6(2): 120–7. 21651748 10.1111/j.1752-699X.2011.00257.x

[25] Garcia-Rio F, Soriano JB, Miravitlles M, Munoz L, Duran-Tauleria E, et al (2011) Overdiagnosing subjects with COPD using the 0.7 fixed ratio: correlation with a poor health-related quality of life. Chest 139(5): 1072–80. 21183609 10.1378/chest.10-1721

[26] Barberà JA, Riverola A, Roca J, Ramirez J, Wagner PD, et al (1994) Pulmonary vascular abnormalities and ventilation-perfusion relationships in mild chronic obstructive pulmonary disease. Am J Respir Crit Care Med 149(2 Pt 1): 423–9. 10.1164/ajrccm.149.2.8306040 8306040

[27] Rodríguez-Roisin R, Drakulovic M, Rodríguez DA, Roca J, Barberà JA, et al (2009) Ventilation-perfusion imbalance and chronic obstructive pulmonary disease staging severity. J Appl Physiol 106(6): 1902–8. 19372303 10.1152/japplphysiol.00085.2009

[28] Ofir D, Laveneziana P, Webb KA, Lam YM, O'Donnell DE (2008) Mechanisms of dyspnea during cycle exercise in symptomatic patients with GOLD stage I chronic obstructive pulmonary disease. Am J Respir Crit Care Med 177(6): 622–9. 18006885 10.1164/rccm.200707-1064OC

[29] Grydeland TB, Dirksen A, Coxson HO, Pillai SG, Sharma S, et al (2009) Quantitative computed tomography: emphysema and airway wall thickness by sex, age and smoking. Eur Respir J 34(4): 858–65. 19324952 10.1183/09031936.00167908

